# Supplementary material for: Harvest year effects on Apulian EVOOs evaluated by 1H NMR based metabolomics
Source: PeerJ. 2016 Dec 15;4:e2740. doi: 10.7717/peerj.2740 (PMC5162422; doi:10.7717/peerj.2740)
Supplement: Supplemental Information 1 [file peerj-04-2740-s001.docx]

**Supplementary information file**

**Harvest year effects on Apulian EVOOs evaluated by ^1^H NMR based metabolomics**

Chiara R. Girelli^‡^, Laura Del Coco^‡^, Paride Papadia, Sandra A. De Pascali, Francesco P. Fanizzi

University of Salento, Di.S.Te.B.A., Campus Ecotekne, via Prov.le Lecce-Monteroni, 73100, Lecce, Italy

^‡^ These authors have contributed equally to this work.

Corresponding Author: Francesco Paolo Fanizzi, Paride Papadia

Campus Ecotekne, via Prov.le Lecce-Monteroni, 73100, Lecce, Italy

e-mail address: fp.fanizzi@unisalento.it; paride.papadia@unisalento.it

**Figure S1**: Annual patterns of cumulative rainfall (millimetres) (indicated as black and white circles respectively for 2013 and 2014 ) and maximum absolute temperatures (T°C) (indicated as black and white boxes respectively for 2013 and 2014) in the meteorological stations: Monte Sant’Angelo (FG), Palo del Colle (BA), Gioia del Colle (BA).

**Figure S2**: Representative spectra for each cultivar (2013-2014 harvesting season), with insets at different magnifications, showing the details that allow to visually differentiate samples. Colors of the cultivars are: Red, Coratina; Green, Cima di Mola; Black, Ogliarola; Blue, Peranzana. Expansions shown with different scales. a) Aldehydic region; b) Aromatic region; c) Vynilic region; d) Bisallylic region;e) Aliphatic chains; f) Full width EVOO spectra.

**Figure S3.** t[1]/t[3] PCA scoreplot made up with 3 out of 4 classes. The exclusion of Coratina class from the PCA model allowed a further separation among the three remaining cultivars, especially for Cima di Mola and Ogliarola cultivars.


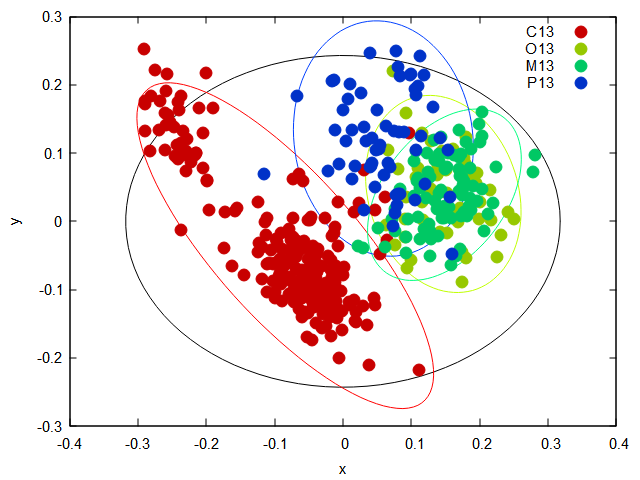


**Figure S4.** *PC1 vs PC2* for the 2013/14 harvest. Ellipses show 95% of explained variance of class.


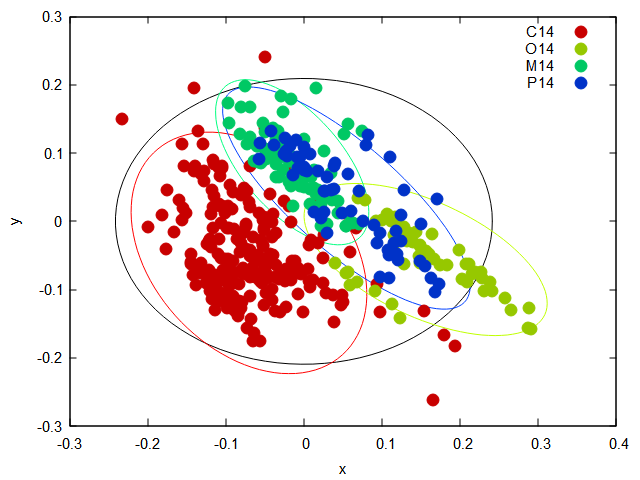


**Figure S5*.*** *PC1 vs PC2* for the 2014/15 harvest. Ellipses show 95% of explained variance of class.

|  | **99% variance (20PCs)** | |
| --- | --- | --- |
| *J_2_ values* | *2013-14* | *2014-15* |
| Cima di Mola | 1.1733E+12 | 3.3511E+15 |
| Coratina | 2.5890E+06 | 2.1799E+05 |
| Ogliarola | 6.0108E+07 | 4.5855E+19 |
| Peranzana | 3.3201E+06 | 5.1181E+12 |

**Table S1.** J_2_ values calculated from the PCA components explaining 99% total variance (20 components).

Coratina

Min. 1st Qu. Median Mean 3rd Qu. Max.

0.004132 0.018090 0.028260 0.043510 0.055570 0.271800

Cima di Mola

Min. 1st Qu. Median Mean 3rd Qu. Max.

0.01370 0.03360 0.04671 0.05097 0.06580 0.15460

Ogliarola

Min. 1st Qu. Median Mean 3rd Qu. Max.

0.009506 0.018600 0.028740 0.037820 0.049320 0.160600

Peranzana

Min. 1st Qu. Median Mean 3rd Qu. Max.

0.009206 0.027680 0.042610 0.049740 0.062130 0.140500

**Table S2.** Summary data for the pairwise Mahalanobis distances for the EVOOs obtained from a single plant in the two successive harvests.
